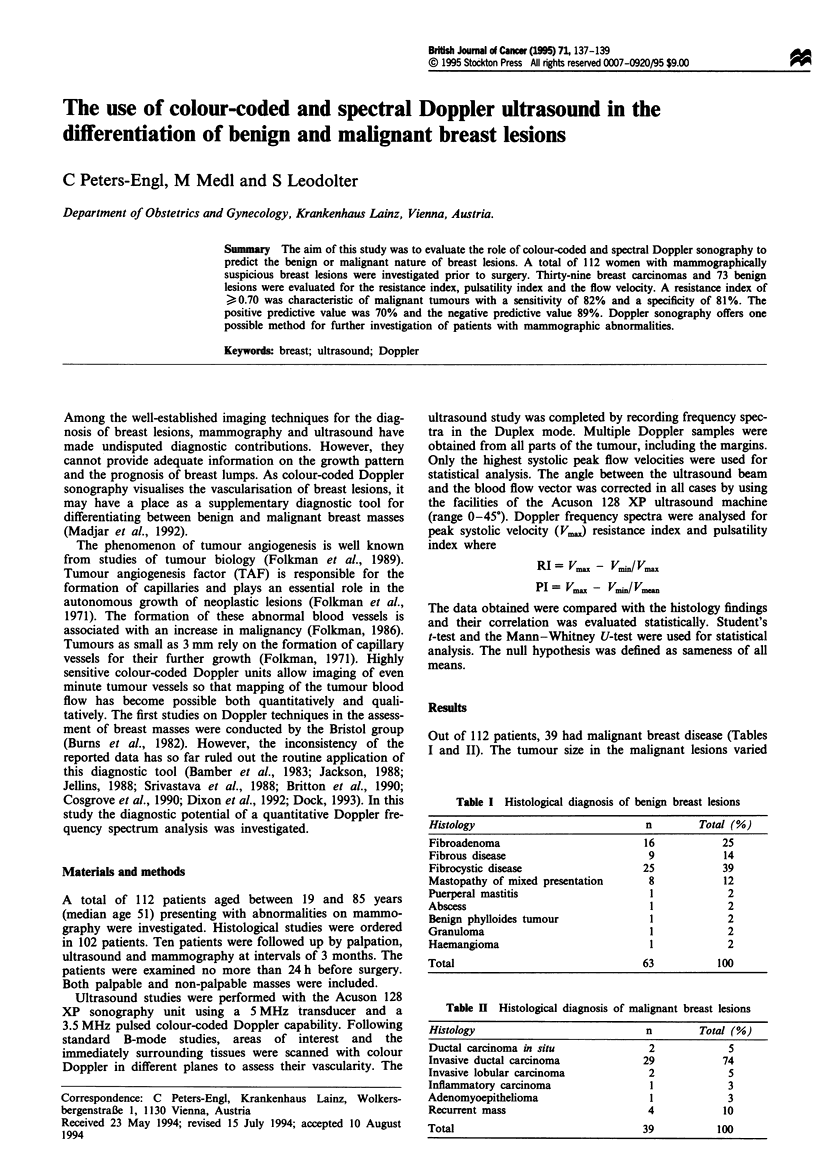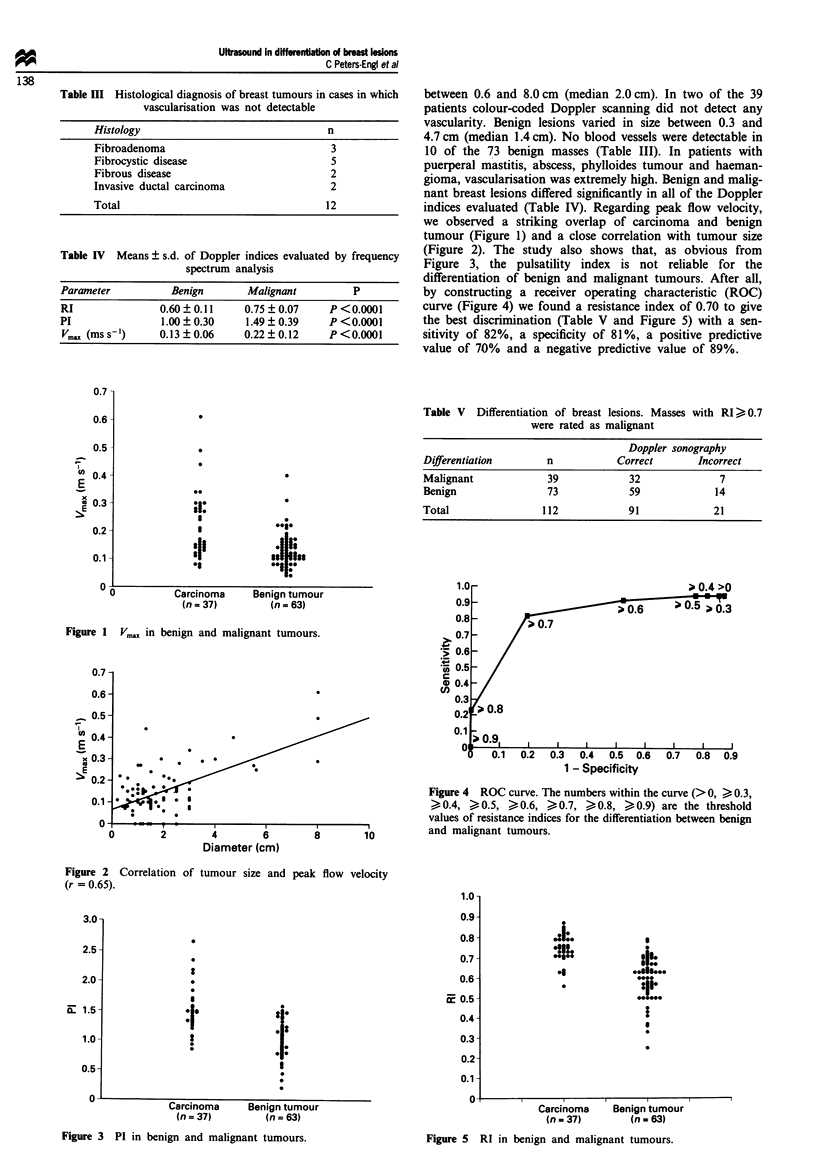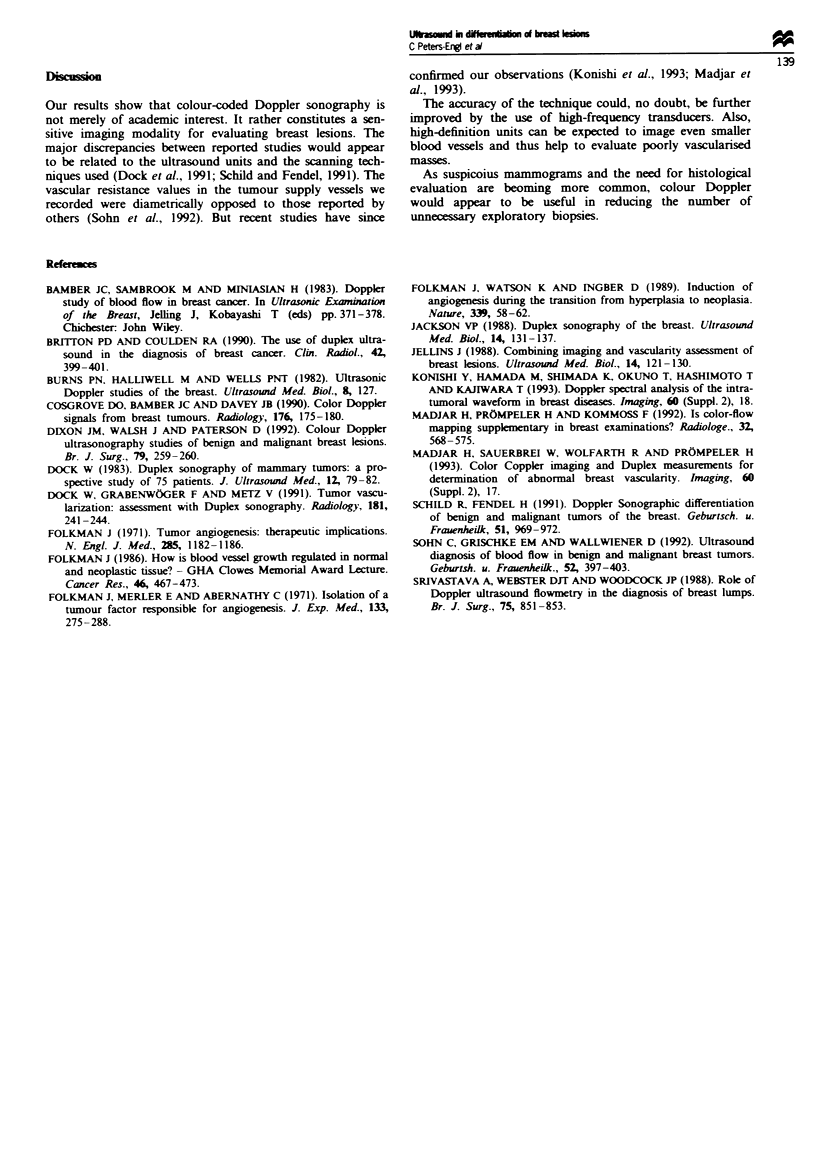# The use of colour-coded and spectral Doppler ultrasound in the differentiation of benign and malignant breast lesions.

**DOI:** 10.1038/bjc.1995.28

**Published:** 1995-01

**Authors:** C. Peters-Engl, M. Medl, S. Leodolter

**Affiliations:** Department of Obstetrics and Gynecology, Krankenhaus Lainz, Vienna, Austria.

## Abstract

The aim of this study was to evaluate the role of colour-coded and spectral Doppler sonography to predict the benign or malignant nature of breast lesions. A total of 112 women with mammographically suspicious breast lesions were investigated prior to surgery. Thirty-nine breast carcinomas and 73 benign lesions were evaluated for the resistance index, pulsatility index and the flow velocity. A resistance index of > or = 0.70 was characteristic of malignant tumours with a sensitivity of 82% and a specificity of 81%. The positive predictive value was 70% and the negative predictive value 89%. Doppler sonography offers one possible method for further investigation of patients with mammographic abnormalities.


© 1995 Stockton Press All rights reserved 0007-0920/95 $9.00

### The use of colour-coded and spectral Doppler ultrasound in the differentiation of benign and malignant breast lesions

C Peters-Engl, M Medl and S Leodolter

*Department of Obstetrics and Gynecology, Krankenhaus Lainz, Vienna, Austria.*

**Summary** The aim of this study was to evaluate the role of colour-coded and spectral Doppler sonography to predict the benign or malignant nature of breast lesions. A total of 112 women with mammographically suspicious breast lesions were investigated prior to surgery. Thirty-nine breast carcinomas and 73 benign lesions were evaluated for the resistance index, pulsatility index and the flow velocity. A resistance index of $\geqslant$0.70 was characteristic of malignant tumours with a sensitivity of 82% and a specificity of 81%. The positive predictive value was 70% and the negative predictive value 89%. Doppler sonography offers one possible method for further investigation of patients with mammographic abnormalities.

**Keywords:** breast; ultrasound; Doppler

Among the well-established imaging techniques for the diagnosis of breast lesions, mammography and ultrasound have made undisputed diagnostic contributions. However, they cannot provide adequate information on the growth pattern and the prognosis of breast lumps. As colour-coded Doppler sonography visualises the vascularisation of breast lesions, it may have a place as a supplementary diagnostic tool for differentiating between benign and malignant breast masses (Madjar *et al.*, 1992).

The phenomenon of tumour angiogenesis is well known from studies of tumour biology (Folkman *et al.*, 1989). Tumour angiogenesis factor (TAF) is responsible for the formation of capillaries and plays an essential role in the autonomous growth of neoplastic lesions (Folkman *et al.*, 1971). The formation of these abnormal blood vessels is associated with an increase in malignancy (Folkman, 1986). Tumours as small as 3 mm rely on the formation of capillary vessels for their further growth (Folkman, 1971). Highly sensitive colour-coded Doppler units allow imaging of even minute tumour vessels so that mapping of the tumour blood flow has become possible both quantitatively and qualitatively. The first studies on Doppler techniques in the assessment of breast masses were conducted by the Bristol group (Burns *et al.*, 1982). However, the inconsistency of the reported data has so far ruled out the routine application of this diagnostic tool (Bamber *et al.*, 1983; Jackson, 1988; Jellins, 1988; Srivastava *et al.*, 1988; Britton *et al.*, 1990; Cosgrove *et al.*, 1990; Dixon *et al.*, 1992; Dock, 1993). In this study the diagnostic potential of a quantitative Doppler frequency spectrum analysis was investigated.

### Materials and methods

A total of 112 patients aged between 19 and 85 years (median age 51) presenting with abnormalities on mammography were investigated. Histological studies were ordered in 102 patients. Ten patients were followed up by palpation, ultrasound and mammography at intervals of 3 months. The patients were examined no more than 24 h before surgery. Both palpable and non-palpable masses were included.

Ultrasound studies were performed with the Acuson 128 XP sonography unit using a 5 MHz transducer and a 3.5 MHz pulsed colour-coded Doppler capability. Following standard B-mode studies, areas of interest and the immediately surrounding tissues were scanned with colour Doppler in different planes to assess their vascularity. The ultrasound study was completed by recording frequency spectra in the Duplex mode. Multiple Doppler samples were obtained from all parts of the tumour, including the margins. Only the highest systolic peak flow velocities were used for statistical analysis. The angle between the ultrasound beam and the blood flow vector was corrected in all cases by using the facilities of the Acuson 128 XP ultrasound machine (range 0-45°). Doppler frequency spectra were analysed for peak systolic velocity ($V_{max}$) resistance index and pulsatility index where

$$RI = V_{max} - V_{min}/V_{max}$$

$$PI = V_{max} - V_{min}/V_{mean}$$

The data obtained were compared with the histology findings and their correlation was evaluated statistically. Student's *t*-test and the Mann-Whitney *U*-test were used for statistical analysis. The null hypothesis was defined as sameness of all means.

### Results

Out of 112 patients, 39 had malignant breast disease (Tables I and II). The tumour size in the malignant lesions varied

**Table I** Histological diagnosis of benign breast lesions

| *Histology* | n | *Total (%)* |
|---|---|---|
| Fibroadenoma | 16 | 25 |
| Fibrous disease | 9 | 14 |
| Fibrocystic disease | 25 | 39 |
| Mastopathy of mixed presentation | 8 | 12 |
| Puerperal mastitis | 1 | 2 |
| Abscess | 1 | 2 |
| Benign phylloides tumour | 1 | 2 |
| Granuloma | 1 | 2 |
| Haemangioma | 1 | 2 |
| Total | 63 | 100 |

**Table II** Histological diagnosis of malignant breast lesions

| *Histology* | n | *Total (%)* |
|---|---|---|
| Ductal carcinoma *in situ* | 2 | 5 |
| Invasive ductal carcinoma | 29 | 74 |
| Invasive lobular carcinoma | 2 | 5 |
| Inflammatory carcinoma | 1 | 3 |
| Adenomyoepithelioma | 1 | 3 |
| Recurrent mass | 4 | 10 |
| Total | 39 | 100 |

Correspondence: C Peters-Engl, Krankenhaus Lainz, Wolkersbergenstraße 1, 1130 Vienna, Austria

Received 23 May 1994; revised 15 July 1994; accepted 10 August 1994

**Table III** Histological diagnosis of breast tumours in cases in which vascularisation was not detectable

| *Histology* | n |
|---|---|
| Fibroadenoma | 3 |
| Fibrocystic disease | 5 |
| Fibrous disease | 2 |
| Invasive ductal carcinoma | 2 |
| Total | 12 |

**Table IV** Means ± s.d. of Doppler indices evaluated by frequency spectrum analysis

| *Parameter* | *Benign* | *Malignant* | P |
|---|---|---|---|
| RI | 0.60 ± 0.11 | 0.75 ± 0.07 | $P < 0.0001$ |
| PI | 1.00 ± 0.30 | 1.49 ± 0.39 | $P < 0.0001$ |
| $V_{max}$ (ms $s^{-1}$) | 0.13 ± 0.06 | 0.22 ± 0.12 | $P < 0.0001$ |

**Figure 1** $V_{max}$ in benign and malignant tumours.

**Figure 2** Correlation of tumour size and peak flow velocity ($r = 0.65$).

**Figure 3** PI in benign and malignant tumours.

between 0.6 and 8.0 cm (median 2.0 cm). In two of the 39 patients colour-coded Doppler scanning did not detect any vascularity. Benign lesions varied in size between 0.3 and 4.7 cm (median 1.4 cm). No blood vessels were detectable in 10 of the 73 benign masses (Table III). In patients with puerperal mastitis, abscess, phylloides tumour and haemangioma, vascularisation was extremely high. Benign and malignant breast lesions differed significantly in all of the Doppler indices evaluated (Table IV). Regarding peak flow velocity, we observed a striking overlap of carcinoma and benign tumour (Figure 1) and a close correlation with tumour size (Figure 2). The study also shows that, as obvious from Figure 3, the pulsatility index is not reliable for the differentiation of benign and malignant tumours. After all, by constructing a receiver operating characteristic (ROC) curve (Figure 4) we found a resistance index of 0.70 to give the best discrimination (Table V and Figure 5) with a sensitivity of 82%, a specificity of 81%, a positive predictive value of 70% and a negative predictive value of 89%.

**Table V** Differentiation of breast lesions. Masses with RI ⩾ 0.7 were rated as malignant

| | | *Doppler sonography* | |
|---|---|---|---|
| *Differentiation* | n | *Correct* | *Incorrect* |
| Malignant | 39 | 32 | 7 |
| Benign | 73 | 59 | 14 |
| Total | 112 | 91 | 21 |

**Figure 4** ROC curve. The numbers within the curve (>0, ⩾0.3, ⩾0.4, ⩾0.5, ⩾0.6, ⩾0.7, ⩾0.8, ⩾0.9) are the threshold values of resistance indices for the differentiation between benign and malignant tumours.

**Figure 5** RI in benign and malignant tumours.

### Discussion

Our results show that colour-coded Doppler sonography is not merely of academic interest. It rather constitutes a sensitive imaging modality for evaluating breast lesions. The major discrepancies between reported studies would appear to be related to the ultrasound units and the scanning techniques used (Dock *et al.*, 1991; Schild and Fendel, 1991). The vascular resistance values in the tumour supply vessels we recorded were diametrically opposed to those reported by others (Sohn *et al.*, 1992). But recent studies have since confirmed our observations (Konishi *et al.*, 1993; Madjar *et al.*, 1993).

The accuracy of the technique could, no doubt, be further improved by the use of high-frequency transducers. Also, high-definition units can be expected to image even smaller blood vessels and thus help to evaluate poorly vascularised masses.

As suspicoius mammograms and the need for histological evaluation are beoming more common, colour Doppler would appear to be useful in reducing the number of unnecessary exploratory biopsies.

### References

BAMBER JC, SAMBROOK M AND MINIASIAN H (1983). Doppler study of blood flow in breast cancer. In *Ultrasonic Examination of the Breast*, Jelling J, Kobayashi T (eds) pp. 371-378. Chichester: John Wiley.

BRITTON PD AND COULDEN RA (1990). The use of duplex ultrasound in the diagnosis of breast cancer. *Clin. Radiol.*, **42**, 399-401.

BURNS PN, HALLIWELL M AND WELLS PNT (1982). Ultrasonic Doppler studies of the breast. *Ultrasound Med. Biol.*, **8**, 127.

COSGROVE DO, BAMBER JC AND DAVEY JB (1990). Color Doppler signals from breast tumours. *Radiology*, **176**, 175-180.

DIXON JM, WALSH J AND PATERSON D (1992). Colour Doppler ultrasonography studies of benign and malignant breast lesions. *Br. J. Surg.*, **79**, 259-260.

DOCK W (1983). Duplex sonography of mammary tumors: a prospective study of 75 patients. *J. Ultrasound Med.*, **12**, 79-82.

DOCK W, GRABENWÖGER F AND METZ V (1991). Tumor vascularization: assessment with Duplex sonography. *Radiology*, **181**, 241-244.

FOLKMAN J (1971). Tumor angiogenesis: therapeutic implications. *N. Engl. J. Med.*, **285**, 1182-1186.

FOLKMAN J (1986). How is blood vessel growth regulated in normal and neoplastic tissue? - GHA Clowes Memorial Award Lecture. *Cancer Res.*, **46**, 467-473.

FOLKMAN J, MERLER E AND ABERNATHY C (1971). Isolation of a tumour factor responsible for angiogenesis. *J. Exp. Med.*, **133**, 275-288.

FOLKMAN J, WATSON K AND INGBER D (1989). Induction of angiogenesis during the transition from hyperplasia to neoplasia. *Nature*, **339**, 58-62.

JACKSON VP (1988). Duplex sonography of the breast. *Ultrasound Med. Biol.*, **14**, 131-137.

JELLINS J (1988). Combining imaging and vascularity assessment of breast lesions. *Ultrasound Med. Biol.*, **14**, 121-130.

KONISHI Y, HAMADA M, SHIMADA K, OKUNO T, HASHIMOTO T AND KAJIWARA T (1993). Doppler spectral analysis of the intratumoral waveform in breast diseases. *Imaging*, **60** (Suppl. 2), 18.

MADJAR H, PRÖMPELER H AND KOMMOSS F (1992). Is color-flow mapping supplementary in breast examinations? *Radiologe.*, **32**, 568-575.

MADJAR H, SAUERBREI W, WOLFARTH R AND PRÖMPELER H (1993). Color Coppler imaging and Duplex measurements for determination of abnormal breast vascularity. *Imaging*, **60** (Suppl. 2), 17.

SCHILD R, FENDEL H (1991). Doppler Sonographic differentiation of benign and malignant tumors of the breast. *Geburtsch. u. Frauenheilk*, **51**, 969-972.

SOHN C, GRISCHKE EM AND WALLWIENER D (1992). Ultrasound diagnosis of blood flow in benign and malignant breast tumors. *Geburtsh. u. Frauenheilk.*, **52**, 397-403.

SRIVASTAVA A, WEBSTER DJT AND WOODCOCK JP (1988). Role of Doppler ultrasound flowmetry in the diagnosis of breast lumps. *Br. J. Surg.*, **75**, 851-853.